# Incorporating a Static Versus Supportive Mobile Phone App Into a Partial Meal Replacement Program With Face-to-Face Support: Randomized Controlled Trial

**DOI:** 10.2196/mhealth.7796

**Published:** 2018-04-18

**Authors:** Emily Brindal, Gilly A Hendrie, Jill Freyne, Manny Noakes

**Affiliations:** ^1^ CSIRO Food and Nutrition Adelaide Australia; ^2^ CSIRO Australian E-Health Research Centre Marsfield Australia

**Keywords:** mHealth, weight loss, diet

## Abstract

**Background:**

Mobile phone apps may be acceptable to users and could improve retention and adherence over more traditional methods, but there is mixed literature supporting their efficacy. In the weight management space, very little is known about how a mobile phone app integrating features beyond text messaging (short message service) can affect behavior, particularly when combined with face-to-face support.

**Objective:**

The objective of this study was to examine the effectiveness of a mobile phone app when combined with a partial meal replacement program including face-to-face support. This paper compares a static versus supportive app over a 6-month randomized trial for effects on weight loss, weight-related biomarkers, and psychological outcomes.

**Methods:**

Overweight and obese adults (71.2% female, 104/146; mean 48.11, SD 11.75 years) were recruited to participate in the weight loss study, and they were randomized on a 1:1 basis using a computer algorithm. The supportive app (n=75) provided information, food intake recording, rewards, prompts for regular interaction through reminders, and the opportunity to review personal compliance with the dietary program. The static app (n=71) included only recipes and weight loss information. Both groups recieved equal amounts of face-to-face support in addition to app.

**Results:**

The overall reduction in app usage over 24 weeks was lower for the supportive app in comparison with the static app; approximately 39.0% (57/146) of the users were still using the app at week 24. Despite the promising results for app usage, there were no differences in weight loss between groups (F1,128.12=0.83, *P*=.36). However, it should be noted that almost 60% (49/84) of all participants lost 5% or more of body weight during the trial. No weight-related biomarkers were significantly different between groups. Both groups experienced an increase in positive mood, but this was significantly higher for those who received the static app (F1,118.12=4.93, *P*=.03).

**Conclusions:**

Although the supportive app was well received by users, we found little evidence of the added benefit of this versus the static app in combination with face-to-face support in a community-delivered weight loss program. Future versions of the app may incorporate more unique behavioral techniques beyond those provided by the consultant to improve the potency of the app.

**Trial Registration:**

Australian New Zealand Clinical Trials Registry ACTRN12613000547741; https://www.anzctr.org.au/Trial/Registration/TrialReview.aspx?id=364187 (Archived by WebCite http://www.webcitation.org/6yivwfMI9)

## Introduction

### Mobile Phones and Weight Loss

There is growing interest in the possible role mobile phones could play in supporting health behavior change [1,2]. A review of literature suggests that text messaging (short message service) could be effective as an adjunct to behavior change interventions [3]. In the domain of weight control, results reported from a year-long study were promising, with close to 3.5 kg higher weight loss in an intervention group receiving mobile support relative to a no intervention control [4].

Mobile phone apps may be acceptable to users and could improve retention and adherence over more traditional methods of weight loss [5], but there is mixed evidence supporting their efficacy [6,7]. A 12-month intervention using a personal digital assistant (PDA) to support a standard weight loss program reported 3.1% more weight loss in the intervention group when compared with a standard care group [8]. Unlike other trials that included no in-person support [6,7], this intervention included face-to-face support in addition to mobile support through the PDA. Other studies also suggest that combining in-person support with technology may be an effective method for delivering weight management programs. Over 30 months, Svetkey et al [9] observed that the effect of technology looked promising during the early stages but described this effect as “transient” with brief, regular personal contact ultimately more effective at assisting participants with sustained weight loss. Therefore, it is unclear whether apps can be a useful adjunct for weight loss interventions when combined with face-to-face or in-person support.

### Combining Mobile Phones With Traditional Methods

Incorporating mobile phone technology with face-to-face contact does potentially minimize cost-effectiveness and reach associated with exclusively technology-driven programs. However, if outcomes can be improved, and the face-to-face contact can be delivered using a method maximizing reach, then this may balance the advantages and disadvantages of both modes of program delivery. A pharmacy environment provides a practical solution as they are readily accessible for a large number of people [10]. Therefore, it was our aim to develop a supportive weight control program that incorporated in-pharmacy delivery through a trained pharmacy assistant as well as a mobile phone app designed to be an adjunct to the wider program by assisting users in monitoring their progress and staying motivated between face-to-face visits. This paper will describe the mobile phone app and the results comparing a supportive versus static app during a 6-month trial of the weight loss program. It is hypothesized that for a group of dieters following a partial meal replacement program including face-to-face support, an interactive and supportive app will be more effective for weight loss than a static app. A partial meal replacement program was chosen as the basis for the weight control program because these diets provide simple dietary prescriptions and demonstrate good weight loss results [11]. At the same time, these programs can also be challenging because they provide little flexibility and limited variety (most meals are in the form of milkshakes). Therefore, the addition of electronic support could have an effect on the overall efficacy of these programs.

## Methods

### Description of the Trial

This study was a 24-week randomized controlled trial (ACTRN12613000547741), including a 12-week active intervention period followed by a 12-week free-living period. The research was approved by the CSIRO Human Research Ethics Committee (Approval 12/14). All participants signed formal consent forms before their participation in this trial.

A detailed description of the method has been published elsewhere [12]. Briefly, participants were asked to follow a partial meal replacement program, and during the initial active period, they received personalized advice from a trained consultant about how to incorporate high-protein meal replacement shakes (manufactured by Probiotec Pty Ltd) and high-protein meals into their lifestyle. Meal replacements were provided for the first 4 weeks, and then the participants were required to purchase them (Aus $1 per sachet) for the remainder of the study period to attempt to better simulate a pharmacy environment. Participants were randomized to one of the 2 groups that received mobile phone apps differing in the number of monitoring tools and supportive features they contained (described in detail below). Both groups received the same level of face-to-face support and the same weight control program. Apps were purpose-designed for the trial and installed manually on the participants’ phones at their first visit.

### Conditions

#### Intervention and Supportive App

The Weight Management Program (WMP) app was designed to support participants’ behavior modification during the partial meal replacement program by providing information, simplifying food intake recording, rewarding positive behavior, and prompting regular interaction through reminders. The features included were selected based on both behavioral theory and successful behavior change techniques, as well as dietetic methods associated specifically with weight loss programs (ie, dietary compliance feedback) and app design features known to improve engagement, such as gamification, through the award of medals.

For purposes of the trial, the prototype WMP was implemented as a native app for iPhones running iOS 6 or later. Upon download, users set up an account entering a username, their starting weight, a weight loss goal, and by when they wish to achieve the goal.

**Figure 1:**
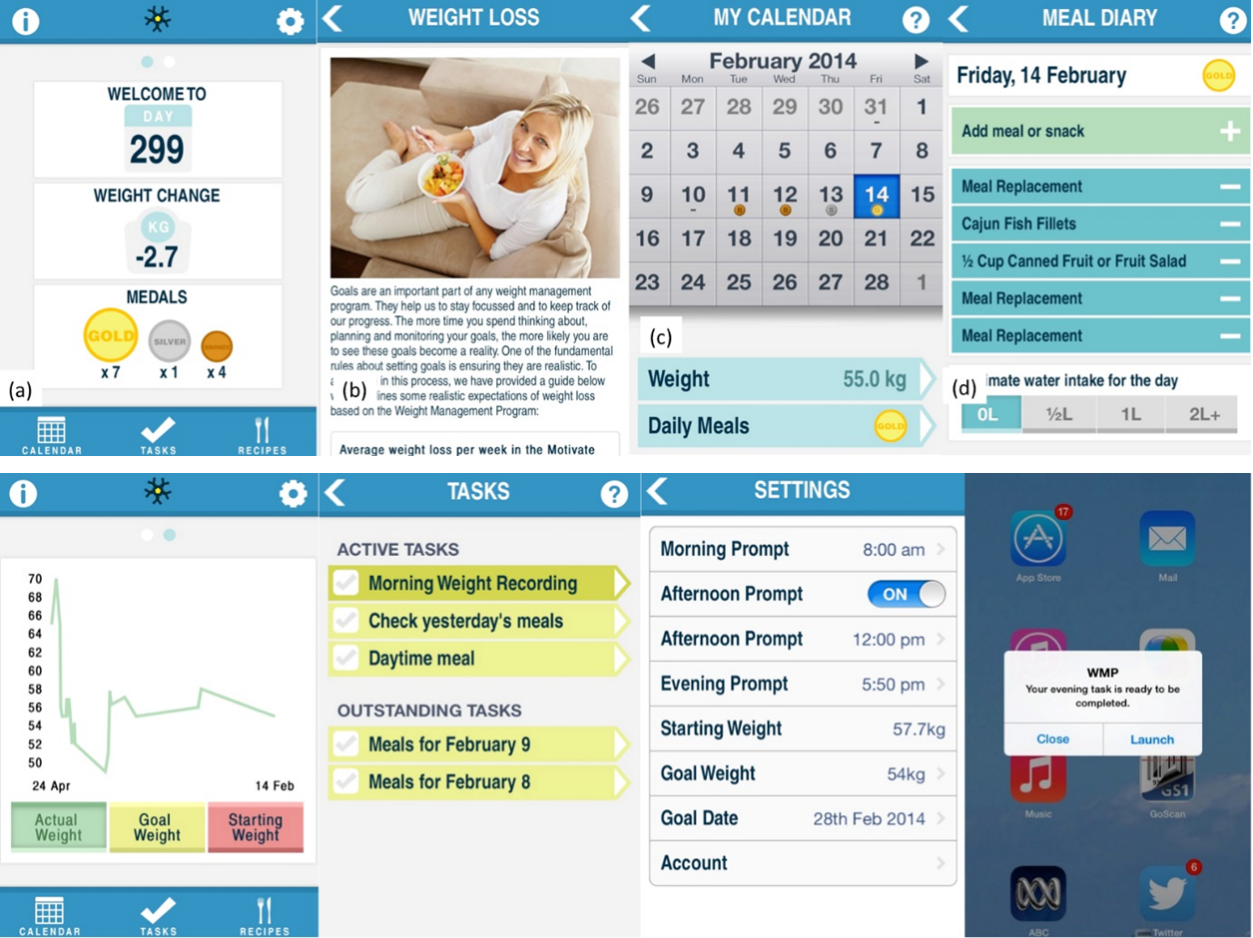
Screenshots of the supportive app showing the Weight Management Program (WMP) home screen; Information; Calendar; Meal diary; Weight loss graph; Task list; Settings; and Push notification.

The WMP home screen included a dashboard access to the tools and services provided in the app (Figure 1). At log-in, each day the users were presented with a randomly selected motivational message or thought for the day on the home screen. Some of these messages were as follows: “Planning ahead will help you to stick to your goals,” “Don't focus on your failures, learn from them,” and “All great achievements take time. Hang in there.” These messages were developed based on the health action process approach of behavior change and included messages to initiate behavior (action-planning) and manage setbacks (coping planning) [13].

The home screen showed a summary of progress, including weight loss and medals received. Dietary information specific to the program was presented in the Information section of the app indicated by the “i” icon on the top left-hand side (Figure 1).

The WMP app provided monitoring tools for weight and food, and it communicated weight loss progress and compliance visually and through virtual rewards. Self-monitoring is considered one of the most effective strategies for behavior change [14]. Recording meals involved the selection of menu items from a list of categories, including Program Meal, Non-program Meal, Meal Replacement, Program Snack, Mini Program Snack, Non-program Snack, and Treat, as outlined in the dietary program. The app included a recipe library of program compliant meals and snacks.

Daily compliance is an essential part of any weight management program. Compliance to the partial meal replacement program was communicated to users through the receipt of gold, silver, and bronze medals, which reflect how well the recorded food intake met the daily guidelines specified by the weight control diet (Figure 1). Medals were also used to add an element of gamification, which has been shown to improve user engagement in other behavioral domains [15]. A gold medal indicated that the guidelines were met, a silver medal indicated that the intake was close to the guidelines, and a bronze medal indicated that some progress toward the guidelines was made. No medal was rewarded if a minimal amount of information was entered, or if a user was well short of the dietary prescriptions. A snapshot of daily intake was shown on the Calendar screen. Users’ weight loss was summarized on the home screen for convenient reflection and presented graphically in a separate section (Figure 1).

The app generated 3 daily task prompts (morning, afternoon, and evening) to encourage self-monitoring. Morning tasks required completion of the meal diary for the previous day and the recording of weight. The afternoon and evening tasks asked the users to update their food diary (Figure 1). Prompt times for tasks were customizable, and afternoon tasks could be disabled by the user. These prompts were all designed to promote closer self-monitoring of progress (weight) and compliance (food diary), and they appeared through push notifications.

#### Control App

The control/static app did not include any recording tools (weight or food) or any tasks. It provided information about the program only, including the detailed recipes. It had the same visual appearance as the home screen on the intervention app with only the recipes button, the day number, and the information button.

### Participants and Outcome Measurements

Overweight and obese adults (aged 18 years and above) were recruited via an established clinic database and local media in Adelaide, South Australia, between March and August 2013. The recruitment process has been published in detail elsewhere [12]. Based on our previous pilot study [6], 61 completers were required to have 80% power to detect a 2.5% difference in weight loss between the 2 groups. To account for participant withdrawals, more than 122 participants were recruited. Based on a drop-out of 20% [6], we aimed to recruit 148 overweight or obese adults. A screening questionnaire was reviewed against eligibility criteria, which included having a body mass index (BMI) greater than 25 kg/m^2^ (based on the self-reported height and weight), access to an iPhone, and willingness to have a pin-prick blood glucose and lipids assessment on 4 occasions at the purpose-built trial clinic. This clinic was designed to replicate a pharmacy environment. On the basis of responses to a medical screener administered by the trial manager, people with known medical conditions, such as diabetes and cancer, were excluded from the study.

#### Objective Outcomes

**Figure 2:**
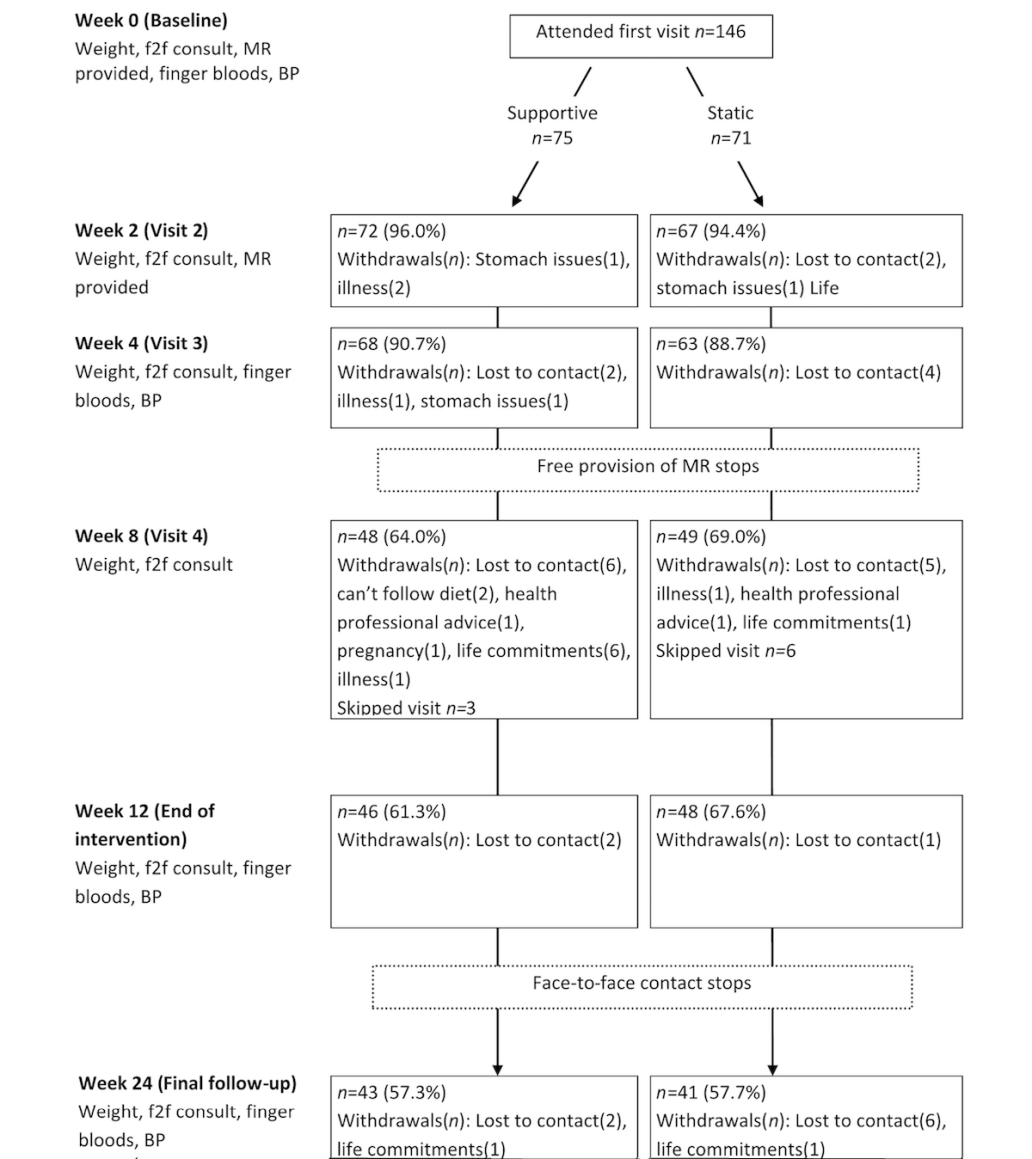
Participant flow diagram. BP: blood pressure; f2f: face-to-face; MR: meal replacement.

The primary outcome measures were percentage weight loss from baseline and changes in blood pressure, fasting blood glucose, and fasting blood lipids (total cholesterol, low-density lipoprotein [LDL], high-density lipoprotein [HDL], and triglycerides). These were measured at baseline; week 2 (weight only); and weeks 4, 12, and 24 (Figure 2). The point-of-care measures were all assessed via a finger prick using AccuCheck devices (Roche Diagnostics Australia, New South Wales, Australia).

#### Psychological Measures

Given the supportive nature of the intervention app, a series of psychological outcomes were included to assess any differences between the 2 apps in terms of changes in mood (positive and negative affect schedule [16]) and stress levels (Perceived Subjective Stress Scale [17]). Given their potential to drive behavior according to the theory of planned behavior, changes in intention and perceived control [18] for continuing the diet program and the intention to continue using the app were also compared between apps.

### App Usage

These data were collected objectively through the logs and database associated with the apps.

### Statistical Methods

All analyses were performed in SPSS version 23 (IBM, Armonk, New York, US). Usage data were aggregated and analyzed using descriptive methods and then compared using general linear models, where appropriate. Mixed models were used to answer the primary hypothesis. These models were designed to assess differences between app condition (main effect) and the interaction between app condition and study week for outcomes, including percentage change from baseline weight, self-reported frequency of weighing, self-reported dietary compliance, and changes in psychological and blood measures (from baseline). All mixed models controlled for participants’ sex, baseline weight (percentage weight change model excepted), and age (in years). Mixed models included all available data and, therefore, were considered an intention-to-treat method of analysis. The numbers presented in the results section are means with standard errors unless otherwise stated. Significance tests were set at *P*<.05. Due to errors in readings, 3 recordings of cholesterol and 1 for blood glucose were deleted from the final analyses and entered as missing values.

### App Bugs During the Trial

Two major technical errors occurred while the trial was underway. Database errors occurred during the first weekend of the trial, which affected only those in the intervention group. Seven users reported problems relating to this. This issue was resolved within 5 days of the initial report. The second technical fault occurred approximately 7-8 weeks after the trial commencement and affected all users. It pertained to an expired enterprise certificate. Five users reported errors relating to this. This fault was resolved within 5 days, and users were asked to reinstall an updated version of the app. They could do this remotely during their visit to the clinic.

## Results

### Participant Description

None of the participant demographics were significantly different between the 2 app groups (Table 1). Most participants were female, had a diploma or a technical certificate, had owned an iPhone for 12 months or longer, and were classed as obesity category 2 according to BMI.

Dropout by the end of week 24 was 42.5% (58/146; Figure 2). There were no differences in attrition between app groups at any week of the study. The greatest dropout occurred after cessation of the provision of free meal replacements. Most dropouts (42%, 24/58) were lost to contact and, therefore, provided no reason for stopping their participation. In total, 14 visits were skipped throughout the trial. This meant that participants returned after missing a visit and, therefore, had missing data for these skipped visits.

### System Usage

**Figure 3:**
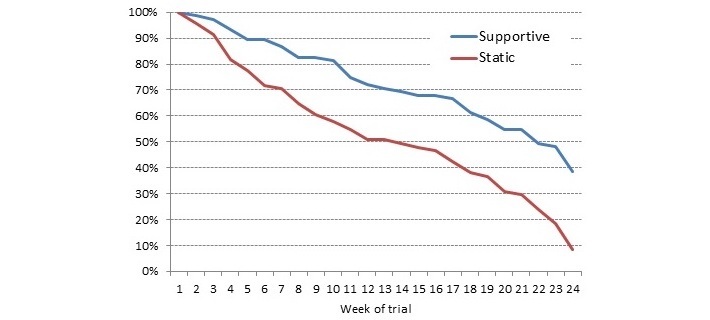
Nonuse attrition of users by app condition throughout the 24 weeks of the trial.

Half of the control group was still using the app at week 12 compared with 72% of those using the intervention app. During the free-living period, usage of the app continued to fall in both groups, with approximately 39% and 9% of the intervention and control groups, respectively, still using the app by the final week of the study (Figure 3). According to univariate analysis of variance, the percentage of days that people interacted with the app (days with interactions/total trial days) was significantly different between groups, with this number higher in the intervention group (43.1%) compared with the control group (11.1%; *F*_1,144_=83.30, *P*<.001).

**Figure 4:**
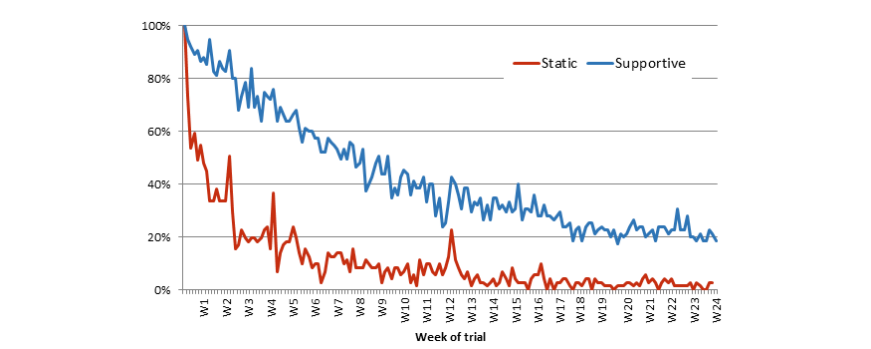
Number of active sample logging in for each day of the trial presented by app condition as a percentage of active (not-withdrawn) users.

**Figure 5:**
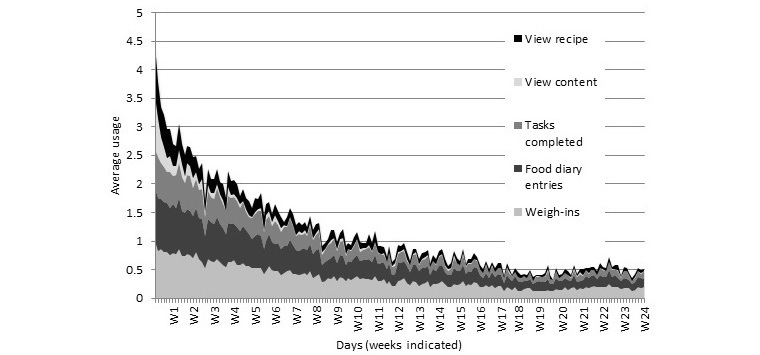
Consumption of different app features for the supportive and intervention app throughout the 24 weeks of the trial.

Negative binomial models suggested that the total number of days an interaction occurred varied significantly between the groups (Wald χ^2^_1,144_=64.9, *P*<.001), with the intervention group having an average of 72.4 (SE 8.4) days and the control group having 18.7 (SE 2.3) days out of a possible 168 days of interaction (Figure 4). The recipes were the central feature of the control app, but the intervention group actually viewed the recipes on more days (23.5 [SE 2.8] vs 8.5 [SE 1.1]; Wald χ^2^_1,144_=35.0, *P*<.001). There was no difference between groups for the number of views of the content providing information on the weight control program, which generally had a low average uptake across the sample (8.90 [SE 0.63]). For the intervention group, the most commonly used features were weight entry and food diary (Figure 5).

**Figure 6:**
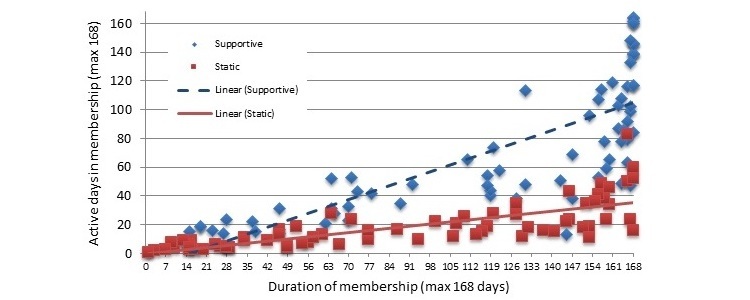
User engagement for both app conditions over the membership duration (number of days between enrollment and last logged use of app).

To compare engagement levels between the 2 apps, the number of views of the weight control program information and the recipes were compared between groups because these were the only actions that appeared in both apps. The number of active days (a day where a recipe- or information-viewing action occurred) was plotted against users’ membership duration (the number of days between enrolment and last logged use of the app). For both groups, there was a positive trend—the longer the membership, the more days with interactions (Figure 6). Users of the supportive app showed higher viewing activity of the recipes and information content.

### Motivation to Use the App

Corresponding to higher usage data, those with the supportive app also had a smaller pooled decrease in their intention to use the app provided (−0.90 [SE 0.22]) relative to those in the control condition (−2.89 [SE 0.21]; *F*_1,113.72_=46.53, *P*<.001). This effect did not interact with week of the trial (*F*_3,90.34_=1.36, *P*=.26).

**Table 1:** Participant demographics and starting characteristics. DPB: diastolic blood pressure; HDL: high-density lipoprotein; LDL: low-density lipoprotein; SBP: systolic blood pressure.

Characteristics		Supportive app (n=75)	Static app (n=71)	Total (n=146)
Sex (female), n (%)		55 (73)	49 (69)	104 (71.2)
Age in years, mean (SE)		48.57 (1.30)	47.76 (1.46)	48.18 (0.98)
**Education, n (%)**				
	Below secondary school	1 (1)	1 (1)	2 (1.4)
	Secondary school	17 (22)	22 (31)	39 (26.7)
	Technical certificate/Diploma	30 (40)	25 (35)	55 (37.7)
	Bachelor's degree	16 (21)	12 (17)	28 (19.2)
	Postgraduate degree	11 (15)	11 (16)	22 (15.1)
Owned phone for >12 months, n (%)		61 (81)	50 (70)	111 (76.0)
**BMI category, n (%)**				
	Overweight (25-30)	15 (20)	12 (17)	27 (18.5)
	Obese category 1 (30-35)	22 (29)	29 (41)	51 (34.9)
	Obese category 2 (35+)	38 (51)	30 (42)	68 (46.6)
**Starting measures, mean (SE)**				
	Weight (kg)	100.68 (2.16)	99.14 (2.38)	99.93 (1.60)
	DPB (mmol/Hg)	80.14 (1.12)	77.84 (1.13)	79.02 (0.80)
	SBP (mmol/Hg)	128.43 (1.70)	127.76 (1.72)	128.10 (1.21)
	Total cholesterol (mmol/L)	4.55 (0.14)	4.90 (0.16)	4.72 (0.11)
	Triglycerides (mmol/L)	1.13 (0.07)	1.20 (0.07)	1.16 (0.05)
	LDL (mmol/L)	2.68 (0.10)	2.92 (0.10)	2.80 (0.07)
	HDL (mmol/L)	1.37 (0.05)	1.36 (0.05)	1.36 (0.04)
	Glucose (mmol/L)	4.78 (0.11)	4.52 (0.12)	4.65 (0.08)

### Percent Weight Change From Baseline

**Figure 7:**
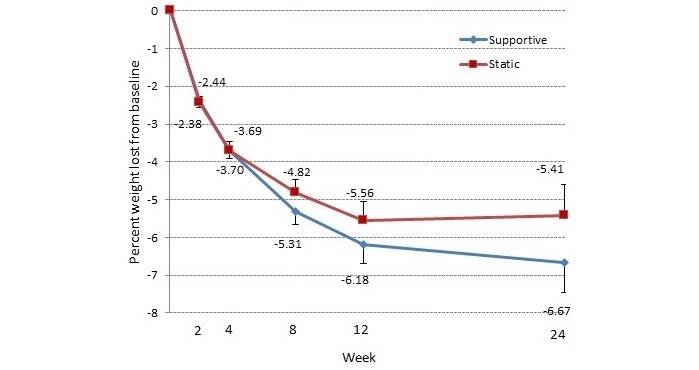
Percentage weight loss from baseline results for each condition presented for each clinic visit throughout the trial. Bars represent 1 standard error.

By week 24, those in the supportive and static app conditions lost 6.67% and 5.41% of their baseline weight, respectively (Figure 7). There were no differences in weight by the different app condition (*F*_1,128.12_=0.83, *P*=.36) or for the interaction between week and app condition (*F*_4,99.94_=0.86, *P*=.49). There was a main effect for sex with males (5.01 [SE 0.32]) losing more weight than females (4.22 [SE 0.26]; *F*_1,135.06_=8.88, *P*=.003). The number of people losing 5% or more of their body weight (a clinically relevant amount of weight) also did not vary by app condition (χ^2^_1,83_=0.2, *P*=.69). Of the 84 completers, 58% (n=49/84) lost 5% or more of their body weight. Those with the supportive app (3.67 [SE 0.10]) reported weighing themselves more frequently than those with the control app (2.90 [SE 0.21]; *F*_1,129.27_=29.74, *P*<.001).

### Dietary Compliance

Perceived dietary compliance (score out of 10) decreased steadily throughout the trial (week 2=9.26 [SE 0.16]; week 4=8.48 [SE 0.19]; week 8=7.47 [SE 0.28]; week 12=7.1.8 [SE 0.27]; and week 24=6.08 [SE 0.28]) but did not vary by app condition (*F*_1,117.84_=0.92, *P*=.34). It was possible to receive dietary compliance feedback daily over the 24-week intervention period (a possible 168 days). Those in the intervention group received some form of dietary compliance feedback (a gold medal, a silver medal, a bronze medal, or no medal) on an average of 76 days. Of all the medals awarded, 26.6% were gold. Interestingly, the number of gold medals received throughout the trial was moderately associated with weight loss at the end of the trial (*r*=.461, *P*<.002).

**Table 2:** Adjusted means for changes in study outcomes for each of the app conditions and results from mixed models for the main effect of treatment and the interaction effect of treatment and study week. DPB: diastolic blood pressure; HDL: high-density lipoprotein; LDL: low-density lipoprotein; SBP: systolic blood pressure.

Measures		Supportive^a^	Static^a^	Treatment		Treatment by week	
		Mean (SE)	Mean (SE)	F (degrees of freedom)	*P* value	F (degrees of freedom)	*P* value
**Blood-related**							
	SBP (mmol/Hg)	−5.81 (1.03)	−5.76 (1.02)	≤ 0.00 (1,109.54)	.97	0.50 (2,95.88)	.61
	DBP (mmol/Hg)	−2.72(0.94)	−2.56 (0.93)	0.02 (1,107.89)	.90	1.88 (2,92.95)	.16
	Total cholesterol (mmol/L)	−0.51 (0.09)	−0.49 (0.09)	0.03 (1,108.75)	.86	0.10 (2,88.81)	.90
	Blood glucose (mmol/L)	−0.07 (0.07)	−0.21 (0.07)	2.34 (1,114.96)	.13	0.44 (2,91.42)	.64
	Triglycerides (mmol/L)	−0.07 (0.04)	−0.13 (0.04)	1.42 (1,104.50)	.24	3.00 (2,81.47)	.06
	LDL (mmol/L)	−0.22 (0.07)	−0.24 (0.07)	0.04 (1,94.44)	.84	0.02 (2,78.28)	.98
	HDL (mmol/L)	−0.14 (0.03)	−0.11 (0.03)	0.45 (1,120.36)	.50	0.42 (2,83.35)	.66
**Psychological**							
	Intention (diet)	−0.77 (0.16)	−0.67 (0.16)	0.23 (1,116.57)	.63	0.80 (3,86.68)	.50
	Behavioral control (diet)	−0.31 (0.09)	−0.07 (0.09)	3.72 (1,99.07)	.06	0.78 (3,91.59)	.51
	Positive affect	0.09 (0.69)	2.17 (0.69)	4.93 (1,118.12)	.03	1.33 (3,92.67)	.27
	Negative affect	−1.61 (0.69)	−0.87 (0.68)	0.64 (1,104.85)	.43	0.73 (3,88.16)	.54
	Weight loss self-efficacy	25.68 (3.1)	23.81 (3.12)	0.20 (1,122.18)	.66	0.99 (3,91.51)	.40
	Subjective stress	−1.08 (0.63)	−1.16 (0.63)	0.01 (1,119.33)	.93	2.35 (3,93.56)	.08

^a^Means are presented with 1 standard error. Means are adjusted for participant age and sex and baseline weight.

### Blood Measures

None of the blood outcomes were significantly different in any of the mixed models (Table 2).

### Psychological Measures

Overall, changes from baseline suggested a consistent decrease in perceived behavioral control and intention to stay on the diet. However, app condition had no differential influence on these outcomes (Table 2). The only psychological measure to be significantly associated with app condition was positive affect. Adjusted mean values suggested that those receiving the static app had a larger increase in positive affect than those with the supportive app.

## Discussion

The aim of this randomized controlled trial was to compare the effect of 2 apps included as part of a weight control program to assess whether a supportive app could improve participant outcomes, including weight, risk factor indicators (such as cholesterol), psychological outcomes (such as mood and motivation), and app engagement. Despite promising results for user engagement (higher usage of the supportive app relative to the static app), we found few differences in the other outcomes assessed between the 2 apps over the 6-month trial.

### Key Findings

The app was one part of a much larger weight control intervention, which also involved face-to-face support and a prescriptive diet program [12]. It may be the case that the additional benefit of the face-to-face contact in the context of the current program limited the ability of the app to have a significant influence on the outcomes assessed. Although positive results have been previously reported using PDAs [8], it is difficult to determine how useful additional mobile phone support is for a variety of styles of weight management programs (calorie counting, group-based, etc).

The apps had significantly different effects on positive affect. Both groups experienced an overall increase in positive affect. However, this was significantly higher for those who were allocated to receive the static app. The direction of this difference was opposite to that seen when comparing similar apps in a previous study [6] and therefore puzzling–especially when paired with objective user data that suggest that those with the static app were not using their app, and subjective reports indicating lower intent to use the app in the control group. Virtual support through apps and other electronic health (eHealth) tools may be the most effective at different stages of behavior change, with face-to-face support being more effective at other times [9]. It may be the case that participants in the static group relied more heavily on the in-person support. All the consultants were trained to provide standard care to each participant. Unfortunately, the amount of face-to-face support that participants received was not recorded or evaluated as part of this trial.

The study retention below 60% and nonuse attrition (less than half still using the app by the end of the study) warrant discussion. We attempted to better replicate a pharmacy environment by including a small cost impediment after an initial weight loss period of 4 weeks. We witnessed a spike in attrition at this point, and this may have inflated our total dropout rate relative to other trials. Including a cost impediment for the meal replacements may have also reduced the weight loss observed as the provision of free products can improve weight outcomes [19]. Fortunately, dropout did not differ between the app groups. Furthermore, the use of intention-to-treat analysis method optimizes statistical power by accounting for missing data. Although nonuse attrition appears high for both of our apps, other studies have seen similar rates in more sophisticated Web-based programs [20]. Indeed, weight management programs, in general, suffer from poor retention and engagement [21].

### Study Strengths and Weaknesses

This study has various strengths that help establish the integrity of its findings. It was a randomized trial, which assessed multiple outcomes through tightly controlled standard operating procedures, and used mostly validated and objective measures. The study also included a variety of outcomes relating directly and indirectly to weight management. Finally, despite witnessing minimal differences between the 2 app conditions, the participants appeared to lose weight, with a majority of completers losing 5% or more of their body weight (a clinically significant amount) by the end of the trial. This suggests that the wider weight control program was successful at promoting weight loss for those retained.

The app targeted specific, evidence-based behavior change techniques considered absent in many commercially available apps [22]. There is little doubt that weight self-monitoring is related to successful weight management [23], and there was a suggestion that the intervention app improved the frequency of weighing. Likewise, the intervention app also successfully targeted diet monitoring—also considered important for weight management [24]. Yet, these behaviors did not translate to observable differences in weight, contrary to previous studies [25]. Weight monitoring may be most effective when combined with feedback [26]. We provided graphical weight summaries to users, but minimal other feedback relating to the weight entries. A future app could target additional behavioral techniques such as contingency planning and problem solving to improve outcomes [27]. Elements of user experience are also likely to improve engagement and, therefore, weight loss. Mining of large amounts of data from a health app also suggests that weight loss success is greater when users can customize features within an app [28]. However, only future controlled trials will reveal the efficacy of these techniques in combination with face-to-face support.

The limitations of this study, such as the restriction to iPhone users, its focus on dietary intervention (more so than exercise) and the primarily female sample have been reported in other similar trials [6] and are unlikely to account for the null effects observed. Although additional features may improve the potency of the supportive app in the future, it remains possible that a supportive app alone is not enough to dramatically influence weight-related outcomes when combined with the support provided in person. Future trials will need to assess the effect of combining multiple forms of support relative to usual care in a community-delivered weight management program.

### Conclusions

We found little evidence of the added benefit of a supportive versus static app in combination with face-to-face support in a clinically delivered weight loss program. Future versions of the app may incorporate more, unique behavioral techniques beyond those provided by the consultant in an effort to improve the potency of the app.

## Abbreviations

BMI: body mass index
DPB: diastolic blood pressure
HDL: high-density lipoprotein
LDL: low-density lipoprotein
PDA: personal digital assistant
SBP: systolic blood pressure
WMP: Weight Management Program

## Appendix

Multimedia Appendix 1 CONSORT-EHEALTH checklist (V 1.6.1).
